# Supplementary material for: Identification of photoperiod-induced specific miRNAs in the adrenal glands of Sunite sheep (Ovis aries)
Source: Front Vet Sci. 2022 Jul 22;9:888207. doi: 10.3389/fvets.2022.888207 (PMC9354845; doi:10.3389/fvets.2022.888207)
Supplement: Supplementary file 1 [file Data_Sheet_1.ZIP › Supplementary file/miRDeep2 file.zip!/miRDeep2 file/Novel_Structure/SP42A1/Novel_68.pdf]

Provisional ID	:	NC_019460.2_6838
Score total	:	35
Score for star read(s)	:	3.9
Score for read counts	:	28.2
Score for mfe	:	1.3
Score for randfold	:	1.6
Score for cons. seed	:	
Total read count	:	67
Mature read count	:	65
Loop read count	:	0
Star read count	:	2

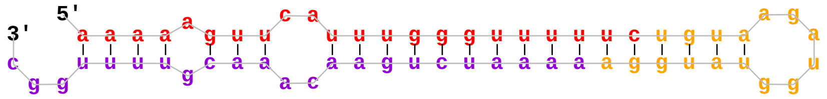

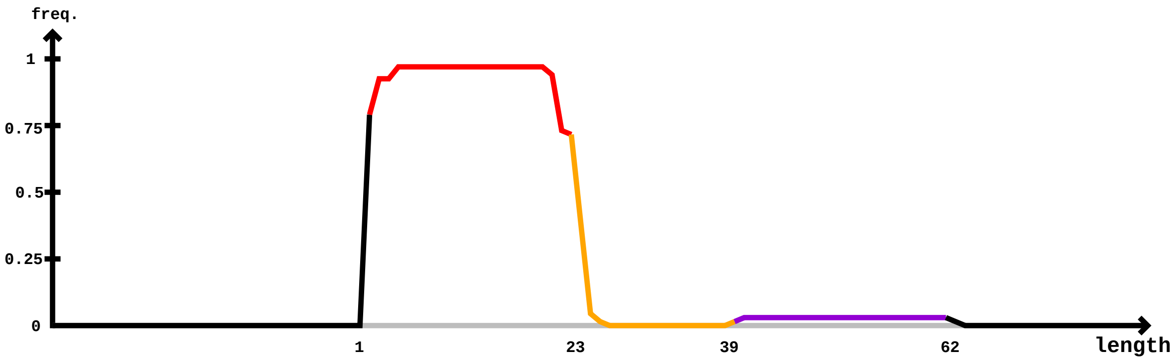


```
                                  Mature                                  Star
5'- gguuccugauugccaauacauuggguuagccaaaaaguucauuuggguuuuucuguaagaugguauggaaaaaucugaacaaacguuuuggccaucaaguauuaggccucug   -3'    obs
    gguuccugauugccaauacauuggguuagccaaaaaguucauuuggguuuuucuguaagaugguauggaaaaaucugaacaaacguuuuggccaucaaguauuaggccucug          exp
    ...........((((((((.((((....(((((((.(((..((((((((((((((((......))))))))))))))))..))).)))))))..))))))))).)))....     reads    mm    sample
    ...............................aaaaaguucGuuuggguuu..............................................................     2        1     seq
    ...............................aaaaaguucGuuuggguuuu.............................................................     12       1     seq
    ...............................aaaaGguucauuuggguuuu.............................................................     1        1     seq
    ...............................aaaaaguucGuuuggguuuuuc...........................................................     38       1     seq
    ................................aaaGguucauuuggguuuu.............................................................     1        1     seq
    ................................Uaaaguucauuuggguuuuu............................................................     1        1     seq
    ................................aaaaguucauuAggguuuuucu..........................................................     6        1     seq
    ................................Uaaaguucauuuggguuuuucu..........................................................     1        1     seq
    ..................................aaguucauuugggCuuuucug.........................................................     2        1     seq
    ..................................aaguucauuuggguuuuucugu........................................................     1        0     seq
    .....................................................................aaaaucugaacaaacUuuuggc.....................     1        1     seq
    ......................................................................aaaucugaacaaacguuuuggc....................     1        0     seq
```
